# Assessment of Sexual Function Following Hysterectomy: A Systematic Review and Meta-Analysis

**DOI:** 10.3390/medsci14030396

**Published:** 2026-07-16

**Authors:** Ákos Molnár-Csendom, Balázs Vida, Ferenc Bánhidy, Francis Bánhidy, Sára Káposzta, Lotti Lőczi, Dániel Sándor Veres, Márton Keszthelyi, Balázs Lintner, Richárd Tóth

**Affiliations:** 1 Department of Obstetrics and Gynecology, Semmelweis University, 1082 Budapest, Hungary; molnar.akos@stud.semmelweis.hu (Á.M.-C.); vida.balazs.lajos@semmelweis.hu (B.V.); banhidy.ferenc@semmelweis.hu (F.B.); kaposzta.sara@stud.semmelweis.hu (S.K.); keszthelyi.lotti.lucia@semmelweis.hu (L.L.); lintner.balazs.zoltan@semmelweis.hu (B.L.); toth.richard@semmelweis.hu (R.T.); 2 Barts Health NHS Trust, The Royal London Hospital, Whitechapel Rd, London E1 1FR, UK; banhidyfp@hotmail.com; 3 Workgroup of Research Management, Doctoral School, Semmelweis University, 1085 Budapest, Hungary; 4 Department of Biophysics and Radiation Biology, Semmelweis University, 1094 Budapest, Hungary; veres.daniel@semmelweis.hu

**Keywords:** hysterectomy, sexual function, Female Sexual Function Index (FSFI), quality of life, cervical preservation, surgical approach

## Abstract

Background: Hysterectomy is one of the most frequently performed major gynecological procedures, yet its effect on sexual quality of life remains unclear. Objective: To evaluate changes in sexual quality of life following hysterectomy and to determine whether cervical preservation is associated with improved postoperative sexual function. Methods: Five databases were systematically searched from inception to February 2026. Eligible studies reported pre- and postoperative sexual quality-of-life outcomes after hysterectomy using validated or non-validated questionnaires. All surgical indications, approaches, and study designs were included. Risk of bias was assessed using RoB 2 and ROBINS-I. Mean score differences were pooled using random-effects meta-analysis and meta-regression within a frequentist framework. Heterogeneity was assessed with τ^2^, cluster-robust standard errors were applied, and certainty of evidence was evaluated using GRADE. Results: Thirty-four studies were included in the systematic review, among them sixteen studies comprising 2341 patients were included in the statistical synthesis comprising three randomized controlled trials and thirteen observational studies. The Female Sexual Function Index (FSFI) was the most commonly reported outcome. Across all hysterectomy types, including total, abdominal, laparoscopic, and vaginal approaches, no clinically or statistically significant improvement in postoperative sexual function was observed compared with baseline. Subtotal hysterectomy did not demonstrate a meaningful advantage over total hysterectomy. FSFI total scores were consistent with sexual dysfunction at baseline and remained below the established cutoff (≤26.55) during follow-up across all surgical routes. No hysterectomy approach was associated with a clinically relevant change in sexual quality of life. Conclusions: Postoperative changes in sexual function after hysterectomy were small and non-significant overall. Cervical preservation did not provide measurable benefit. Surgical approach selection should therefore not be based on expectations of improved sexual outcomes.

## 1. Introduction

Hysterectomy is among the most frequently performed major gynecologic procedures worldwide and remains a cornerstone in the management of both benign and malignant gynecologic conditions [1]. The procedure involves partial or complete removal of the uterus and, through its anatomical and psychosocial consequences, may influence postoperative quality of life, particularly sexual well-being. Despite its widespread use, the impact of hysterectomy on women’s sexual quality of life remains incompletely characterized.

Quality of life (QoL) is a multidimensional construct encompassing individuals’ subjective perceptions of physical, psychological, and social functioning. Within this framework, sexual quality of life represents a clinically important and sensitive domain for women undergoing hysterectomy. It includes sexual function, covering physiological domains such as desire, arousal, orgasm, and pain, as well as sexual satisfaction, which reflects subjective experiences of pleasure, fulfilment, and concordance with personal expectations [2].

Hysterectomy may be classified according to both the extent of uterine resection and the surgical approach. Based on anatomical extent, procedures include total hysterectomy, which involves removal of the uterine corpus and cervix; subtotal (supracervical) hysterectomy, in which the cervix is preserved; and radical hysterectomy, which entails excision of the uterus, cervix, upper vagina, and parametrial tissues. With respect to surgical approach, hysterectomy may be performed via an abdominal or vaginal route or by using minimally invasive techniques such as laparoscopy or robot-assisted surgery. These variations may differentially affect postoperative sexual outcomes through their influence on pelvic anatomy, neural integrity, and postoperative recovery [3].

Several validated instruments are available to assess sexual quality of life in women. Among the most widely used are the Female Sexual Function Index (FSFI) and the Arizona Sexual Experiences Scale (ASEX). The FSFI is the most commonly used instrument and evaluates sexual function across six domains: desire, arousal, lubrication, orgasm, satisfaction, and pain, using 19 items, with higher scores indicating better sexual function. The maximum score is 36 points, the established clinical cutoff score for sexual dysfunction is defined as a score below 26.55. Although a universally accepted minimally clinically important difference (MCID) for the FSFI has not been definitively established, previous studies have suggested that an improvement of approximately 3–4.2 points may represent a clinically meaningful change in total FSFI score [4,5]. In contrast, the ASEX is a brief, five-item questionnaire assessing core aspects of sexual functioning, in which higher scores indicate greater sexual dysfunction [6]. Other questionnaires include the Pelvic Organ Prolapse/Urinary Incontinence Sexual Questionnaire (PISQ-12), which assesses sexual function with urinary symptoms, and the McCoy Female Sexuality Questionnaire (MFSQ) a revised instrument used to evaluate changes in female sexual function associated with menopausal hormone changes [7,8]. The Golombok–Rust Inventory of Sexual Satisfaction (GRISS) is a self-report questionnaire that consists of 28 items and evaluates sexual dysfunction among women and men as well [9]. Tübinger scale for sexual therapy (TSST) is a German language test used to assess women’s sexual behaviour. These instruments are used less frequently than FSFI and ASEX. The use of heterogeneous instruments and outcome definitions has contributed to variability and inconsistency in reported findings across studies.

Previous systematic reviews often pooled the results from different questionnaires despite their difference in structure and scoring system, which may contribute to inconsistent findings. Therefore, a comprehensive evaluation of postoperative sexual outcomes using questionnaire-specific analyses and stratification by surgical approach may provide a more detailed understanding of the impact of hysterectomy on sexual function.

## 2. Materials and Methods

This systematic review and meta-analysis was conducted in accordance with the Preferred Reporting Items for Systematic Reviews and Meta-Analyses (PRISMA) 2020 guidelines (Supplementary Table S1) and the recommendations of the Cochrane Handbook for Systematic Reviews of Interventions. The study protocol was prospectively registered with PROSPERO (PROSPERO 2025 CRD420251275411), and the review was conducted in accordance with the registered protocol.

### 2.1. Eligibility Criteria

The eligibility criteria were defined a priori using the population–intervention–comparison–outcome (PICO) framework. The population comprised adult women undergoing hysterectomy for benign or malignant indications. Eligible surgical interventions included total abdominal hysterectomy, total laparoscopic hysterectomy, vaginal hysterectomy, supracervical (subtotal) hysterectomy, and radical hysterectomy. Comparisons were made between hysterectomy types where data were available. The primary outcome was change in sexual function measured using validated patient-reported outcome questionnaires. These included the Female Sexual Function Index (FSFI), Arizona Sexual Experiences Scale (ASEX), Pelvic Organ Prolapse/Urinary Incontinence Sexual Questionnaire 12 (PISQ-12), McCoy Female Sexuality Questionnaire (MFSQ), Golombok–Rust Inventory of Sexual Satisfaction (GRISS), and the Tübinger scale for sexual therapy (TSST). Outcomes were extracted as changes in total scores and, where reported, domain-specific subscale scores.

Studies employing non-validated sexual function questionnaires were included in the qualitative synthesis but excluded from quantitative analysis due to the lack of established psychometric comparability.

To minimize clinical heterogeneity, only studies evaluating hysterectomy without concomitant bilateral salpingo-oophorectomy (BSO) were considered eligible for quantitative synthesis. Studies including BSO were retained for qualitative analysis when relevant but were excluded from the meta-analysis due to the substantial endocrine effects associated with ovarian removal. Similarly, studies evaluating radical hysterectomy were included only in the qualitative synthesis only and were excluded from quantitative analysis because of their distinct surgical extent and oncologic context.

### 2.2. Information Sources

A comprehensive literature search was conducted in February 2026 using PubMed/MEDLINE, Web of Science, Scopus, EMBASE, and the Cochrane Central Register of Controlled Trials (CENTRAL). Searches were performed from database inception without restrictions on publication date or study status and were limited to English-language articles. Conference abstracts, preprints, and unpublished studies were excluded. Reference lists of all included studies and relevant review articles were manually screened to identify additional eligible publications.

### 2.3. Search Strategy

A comprehensive literature search was conducted using free-text terms to find all studies relevant to our topic. Search keys in relation to sexual function and dysfunction (e.g., sexual dysfunction, sexual function, dyspareunia) were combined with terms related to hysterectomy (vaginal hysterectomy, total hysterectomy) using the Boolean operator AND. The complete search key set can be found in the Supplementary Material.

### 2.4. Study Selection

All retrieved records were imported into EndNote 25 (Clarivate Analytics, Philadelphia, PA, USA) for reference management and duplicate removal. Study selection was conducted using the Rayyan web-based systematic review platform (Rayyan Systems Inc., Cambridge, MA, USA). Two reviewers (Á.M.-Cs. and B.V.) independently screened titles and abstracts, followed by full-text assessment of potentially eligible articles. Disagreements were resolved through discussion, with consultation of a third reviewer (M.K.) when consensus could not be reached. Inter-reviewer agreement was assessed using Cohen’s kappa statistic.

### 2.5. Data Extraction

Extracted data included study characteristics (first author, year of publication, and study design), sample size, patient characteristics (mean or median age and body mass index), hysterectomy type, ovarian status, sexual function questionnaire used, timing of postoperative assessment, and preoperative and postoperative sexual function outcomes.

Sexual function data were extracted as reported in the original studies, including total scores and domain-specific subscale scores where available, together with corresponding measures of dispersion. Discrepancies between reviewers were resolved through discussion and verification against the original publications (Table 1).

### 2.6. Risk of Bias Assessment

Risk of bias was independently assessed by two reviewers for studies reporting sexual function outcomes using validated questionnaires. Randomized controlled trials were evaluated using the Cochrane Risk of Bias 2 (RoB 2) tool, assessing bias arising from the randomization process, deviations from intended interventions, missing outcome data, outcome measurement, and selective reporting. Non-randomized studies were assessed using the Risk Of Bias In Non-randomized Studies of Interventions (ROBINS-I) tool, which evaluates bias due to confounding, participant selection, intervention classification, deviations from intended interventions, missing data, outcome measurement, and selective reporting. Disagreements were resolved through discussion with a third reviewer.

### 2.7. Data Synthesis

The primary effect size was defined as the mean change in sexual function questionnaire scores. Meta-analyses were conducted within a frequentist framework in accordance with the methodological guidance of Harrer et al. and Viechtbauer. Given the anticipated clinical and methodological heterogeneity across studies, random-effects models were applied throughout [10,11]. Hysterectomy types were treated as distinct surgical exposures and were not pooled across groups. Quantitative analyses were performed separately for each hysterectomy type and outcome measure when sufficient data were available. When fewer than two studies reported comparable outcomes for a given hysterectomy type, quantitative synthesis was not performed, and findings were summarized narratively. Several studies reported sexual function outcomes at multiple postoperative time points. To account for within-study correlation between repeated measurements in the same patient cohort, meta-regression models were applied. The outcome variable was the reported questionnaire score, with time since surgery modelled as a continuous variable with a linear effect. Descriptive forest plots were generated to display individual study effect sizes with corresponding 95% confidence intervals. Meta-regression results are presented in tabular form and as scatterplots. Funnel plots were constructed to illustrate the relationship between effect size estimates and their sampling error; however, formal assessment of publication bias was limited by heterogeneity in follow-up time points across studies. All statistical analyses were performed using R software (version 4.5.1). Model estimation was conducted using the metafor package (version 4.8.0). Cluster-robust variance estimation was implemented using the clubSandwich package (version 4.8.0). Standardized mean difference calculations and forest plots were generated using the meta package (version 8.0.2), and graphical outputs were created with ggplot2 (version 4.0.0). For meta-regression analyses, variance components were estimated using restricted maximum likelihood (REML). A multivariate model was specified to account for the dependence among effect size estimates within studies, including correlations arising from both sampling errors and shared underlying true effects. Baseline measurements were defined as time zero. A continuous-time autoregressive (CAR) structure was used to model the correlation between repeated measurements within studies, with the correlation decreasing as a function of the time interval between observations. For studies with only two measurements, the CAR structure reduces to a conventional specification of a single correlation between the two observations. The autocorrelation parameter (φ) was set at 0.2. When only two time points were available, this approach is equivalent to assuming a fixed pre–post correlation coefficient (r). Sensitivity analyses were performed by varying φ from 0.1 to 0.9 in increments of 0.1, with no meaningful changes observed in the estimated intercepts or slopes. Model diagnostics included inspection of standardized residuals versus fitted values and quantile–quantile plots for standardized residuals and random effects. Based on these diagnostics, model assumptions were considered acceptable. To improve the robustness of statistical inference, “CR2” cluster-robust standard errors and small-sample corrected inference were applied using study as the clustering unit. As an exploratory analysis, age was included as an additional explanatory variable without interaction with time, due to the limited number of studies and sample sizes available.

### 2.8. Confidence in Cumulative Evidence

The certainty of evidence for each primary and secondary sexual function outcome was assessed using the Grading of Recommendations, Assessment, Development, and Evaluation (GRADE) framework. This approach evaluates five domains: risk of bias, inconsistency, indirectness, imprecision, and publication bias. Based on these domains, the certainty of evidence was classified as high, moderate, low, or very low. Randomized controlled trials were initially rated as high-certainty evidence and were downgraded when methodological limitations were identified in one or more domains. Non-randomized studies were initially rated as low-certainty evidence and were eligible for upgrading in the presence of a large magnitude of effect, evidence of a dose–response relationship, or when all plausible residual confounding would be expected to attenuate the observed effect. Summary of Findings tables were generated using GRADEpro GDT: GRADEpro Guideline Development Tool [Software]. McMaster University and Evidence Prime, 2023. Available from https://www.gradepro.org/. to present pooled effect estimates for key sexual function outcomes alongside corresponding certainty ratings. All decisions to downgrade or upgrade the certainty of evidence were prespecified and explicitly justified.

### 2.9. Ethical Statement

As this study did not involve the participation of patients or members of the public, and no original data were collected, formal ethical approval was not required. The research was based solely on previously published studies and publicly available data, which eliminates any direct interaction with human participants.

## 3. Results

### 3.1. Study Selection

**Figure 1:**
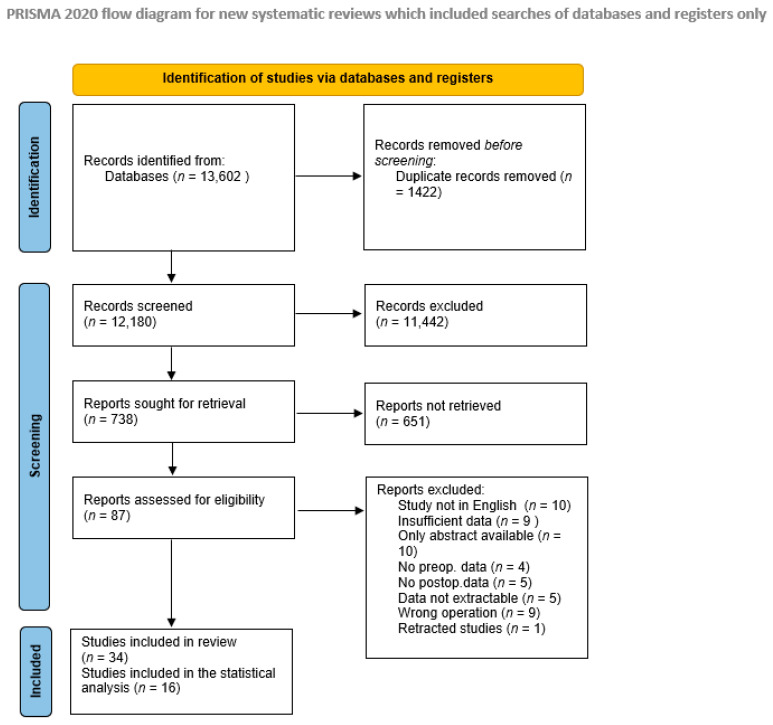
The PRISMA flowchart of the included studies.

The database search identified 13,602 records. After duplicate removal and title and abstract screening, 12,864 records were excluded. Of the remaining 738 articles assessed in full text, 651 were excluded for not meeting the eligibility criteria. Eighty-seven studies underwent a detailed eligibility assessment, of which 48 were excluded. Ultimately, 39 studies met the inclusion criteria and were included in the systematic review (Figure 1).

Of these 34 studies, 16 were eligible for quantitative synthesis, comprising a total of 2341 patients. The remaining 18 studies were excluded from meta-analysis due to substantial methodological and clinical heterogeneity. Specifically, these studies differed with respect to the sexual function questionnaire used, hysterectomy type, ovarian status, and/or the reporting of postoperative follow-up time points, resulting in combinations that could not be meaningfully synthesized statistically.

Although radical hysterectomy was prespecified as an eligible intervention, the number of studies reporting sexual function outcomes following radical hysterectomy was insufficient to permit quantitative analysis. These studies were therefore included in the qualitative synthesis only. Similarly, studies using questionnaires other than FSFI or ASEX (including PISQ-12, MFSQ, TSST, GRISS, and non-validated instruments), studies that did not specify hysterectomy type, and studies involving concomitant bilateral salpingo-oophorectomy (BSO) were summarized narratively.

The final quantitative analyses therefore comprised a relatively homogeneous subset of studies, predominantly evaluating benign indications for hysterectomy, reporting sexual function using the FSFI or ASEX, clearly specifying hysterectomy type, and excluding concomitant BSO (Figure 1).

### 3.2. Study Characteristics

The Female Sexual Function Index (FSFI) was the most frequently used instrument for assessing sexual function and was reported in all 16 studies included in the quantitative synthesis [12,13,14,15,16,17,18,19,20,21,22,23,24,25,26,27]. The Arizona Sexual Experiences Scale (ASEX) was used in three studies and was analyzed separately [18,20,22]. Other validated sexual function questionnaires were employed too infrequently to permit quantitative synthesis. In six studies, the interval between surgery and postoperative assessment was not explicitly reported. These studies were analyzed separately from those with clearly defined follow-up time points.

### 3.3. Risk of Bias of Included Studies

Risk of bias was assessed separately for randomized and non-randomized studies using validated tools. All randomized controlled trials were evaluated using the Cochrane Risk of Bias 2 (RoB 2) tool and were judged to have a low risk of bias across assessed domains. Non-randomized studies were assessed using the Risk Of Bias In Non-randomized Studies of Interventions (ROBINS-I) tool and were judged to be at moderate risk of bias, primarily due to potential confounding and limitations inherent to observational study designs.

### 3.4. Laparoscopic Hysterectomy

#### 3.4.1. Total Laparoscopic Hysterectomy with Defined Follow-Up Time Points

**Figure 2:**
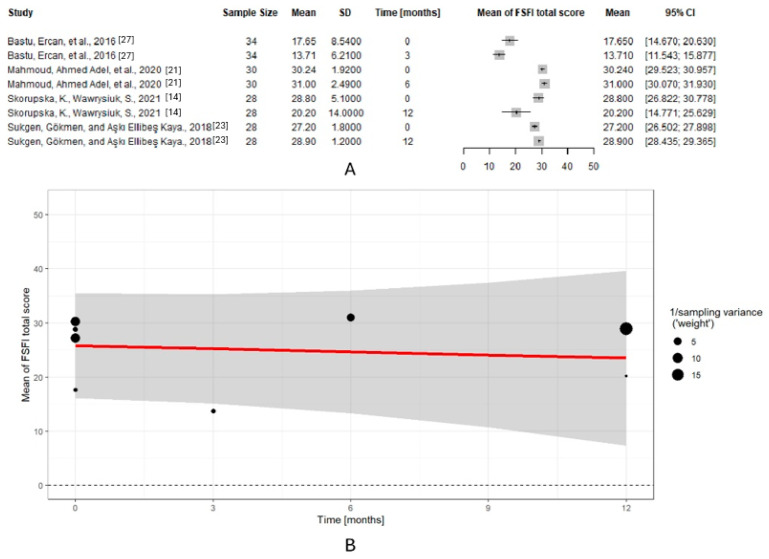
Changes in sexual quality of life following TLH, after defined follow-up time points. (**A**) Forest plot presenting study-specific mean FSFI total scores at predefined postoperative follow-up time points (in months), with corresponding 95% confidence intervals. Squares represent observed mean values, and horizontal lines indicate confidence intervals. (**B**) Bubble plot illustrating the temporal trajectory of FSFI total scores across follow-up. Each bubble represents a study-specific mean at a given time point, with bubble size proportional to study. The red line depicts the estimated pooled trend over time, and the shaded area represents the 95% confidence interval [14,21,23,27].

For total laparoscopic hysterectomy (TLH) with precisely defined follow-up intervals, the pooled mean baseline FSFI total score was 25.08 (95% CI, 16.1339–35.4726; *p* = 0.034). The pooled mean change in FSFI total score over time was 0.1921 points (95% CI, −1.3507–0.9665; *p* = 0.57), indicating no statistically significant change in sexual function following surgery. TLH with clearly defined follow-up intervals was assessed in four articles [14,21,23,27] (Figure 2).

#### 3.4.2. Total Laparoscopic Hysterectomy Without Defined Follow-Up Time Points

**Figure 3:**
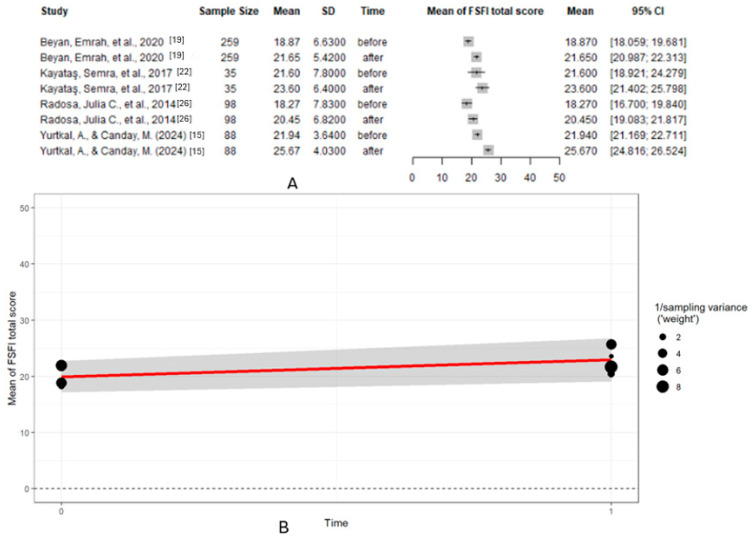
Change in sexual quality of life following TLH. (**A**) Forest plot presenting study-specific mean FSFI total scores before and after TLH, with corresponding 95% confidence intervals. (**B**) Bubble plot illustrating the pooled change in FSFI total score over time (0 = preoperative, 1 = postoperative). Bubble size is proportional to study weight (inverse sampling variance). The red line represents the estimated pooled effect, and the shaded area indicates the 95% confidence interval [15,19,22,26].

For total laparoscopic hysterectomy (TLH), the pooled mean baseline FSFI total score was 19.94 (95% CI, 17.1598–22.7291; *p* = 0.0002). The pooled mean change in FSFI total score over follow-up was 2.9874 points (95% CI, 1.3374–4.6375; *p* = 0.0161), indicating no statistically significant postoperative change in sexual function. TLH without precisely defined follow-up intervals was assessed in four studies [15,19,22,26] (Figure 3).

### 3.5. Total Abdominal Hysterectomy Without Defined Follow-Up Time Points

**Figure 4:**
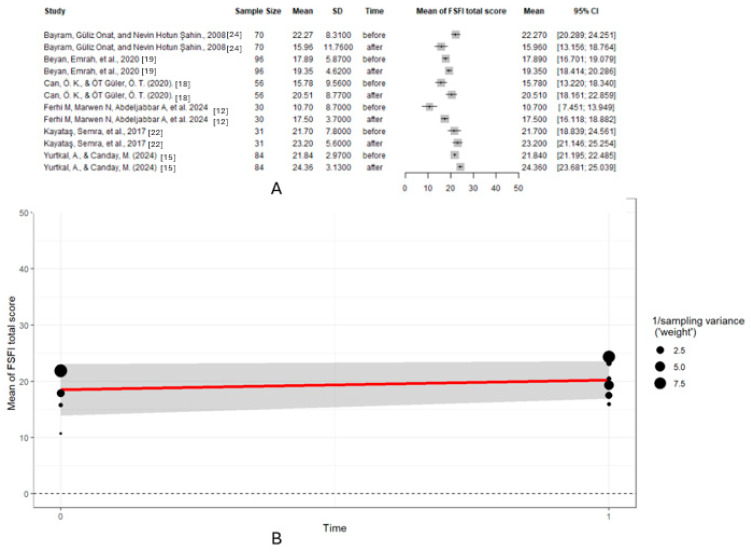
Change in sexual quality of life following TAH. (**A**) Forest plot presenting study-specific mean FSFI total scores before and after TAH, with corresponding 95% confidence intervals. (**B**) Bubble plot illustrating the pooled change in FSFI total score over time (0 = preoperative, 1 = postoperative). Bubble size is proportional to study weight (inverse sampling variance). The red line represents the estimated pooled effect, and the shaded area indicates the 95% confidence interval [12,15,18,19,22,24].

Abdominal hysterectomy (TAH) was assessed in six studies, the pooled mean baseline FSFI total score was 18.52 (95% CI, 13.9361–23.0947; *p* < 0.0001). The pooled mean change in FSFI total score over time was 1.73 points (95% CI −2.66–6.11; *p* = 0.36), indicating no statistically significant postoperative change in sexual function. [12,15,18,19,22,23] (Figure 4).

#### 3.5.1. Total Abdominal Hysterectomy with Defined Follow-Up Time Points

**Figure 5:**
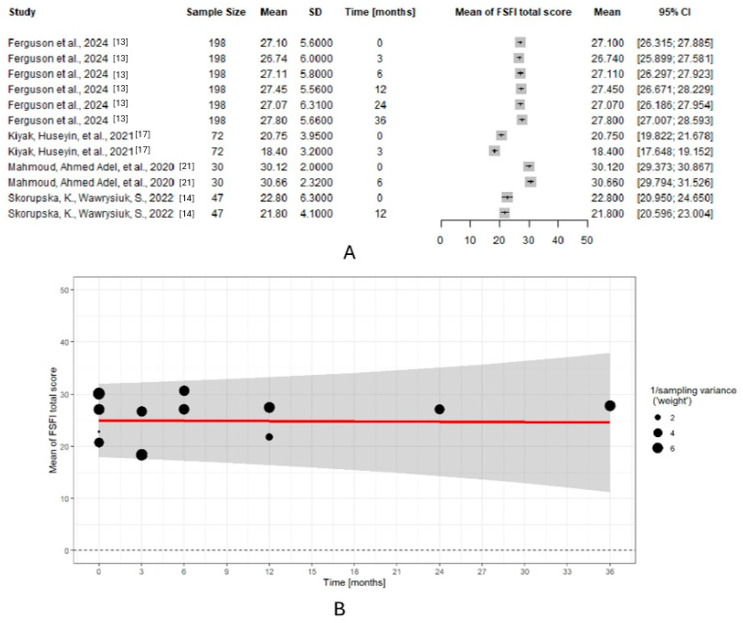
Changes in sexual quality of life following TAH, after defined follow-up time points. (**A**) Forest plot presenting study-specific mean FSFI total scores at predefined postoperative follow-up time points (in months), with corresponding 95% confidence intervals. Squares represent observed mean values, and horizontal lines indicate confidence intervals. (**B**) Bubble plot illustrating the temporal trajectory of FSFI total scores across follow-up. Each bubble represents a study-specific mean at a given time point, with bubble size proportional to study. The red line depicts the estimated pooled trend over time, and the shaded area represents the 95% confidence interval [13,14,17,21].

For total abdominal hysterectomy (TAH) with clearly defined follow-up intervals, the pooled mean baseline FSFI total score was 24.98 (95% CI, 17.9568–32.0106; *p* = 0.015). The pooled mean change in FSFI total score over time was −0.0013 points (95% CI, −0.3789–0.3563; *p* = 0.87), indicating no statistically significant change in sexual function following surgery. TAH with precisely defined follow-up intervals was assessed in four studies [13,14,17,21] (Figure 5).

#### 3.5.2. Total Abdominal Hysterectomy Assessed with ASEX

**Figure 6:**
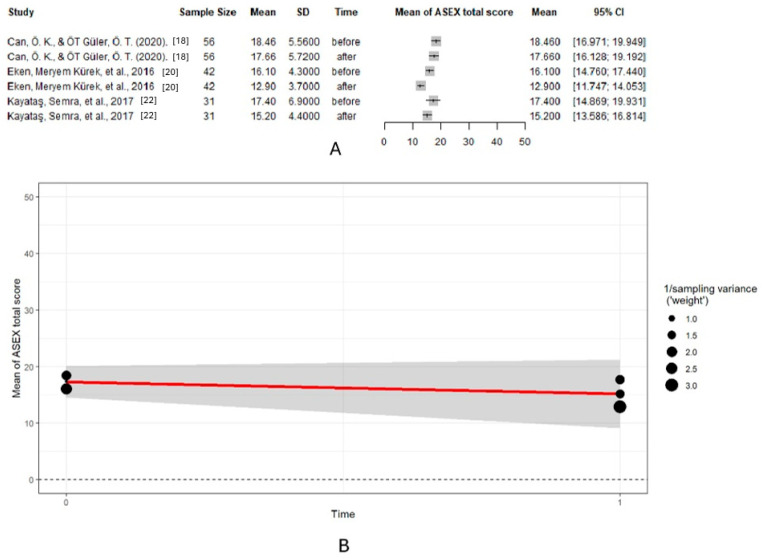
Change in sexual quality of life following TAH. (**A**) Forest plot presenting study-specific mean ASEX total scores before and after TAH, with corresponding 95% confidence intervals. (**B**) Bubble plot illustrating the pooled change in ASEX total score over time (0 = preoperative, 1 = postoperative). Bubble size is proportional to study weight (inverse sampling variance). The red line represents the estimated pooled effect, and the shaded area indicates the 95% confidence interval [18,20,22].

For total abdominal hysterectomy (TAH), the pooled mean baseline ASEX total score was 17.32 (95% CI, 14.5378–20.0934; *p* = 0.0014). The pooled mean change in ASEX total score after follow-up was 2.14 (95% CI, −5.6292–1.346; *p* = 0.1147) indicating no statistically significant postoperative change in sexual function. TAH without defined follow-up intervals assessed using ASEX was reported on in three studies [18,20,22] (Figure 6).

### 3.6. Vaginal Hysterectomy

**Figure 7:**
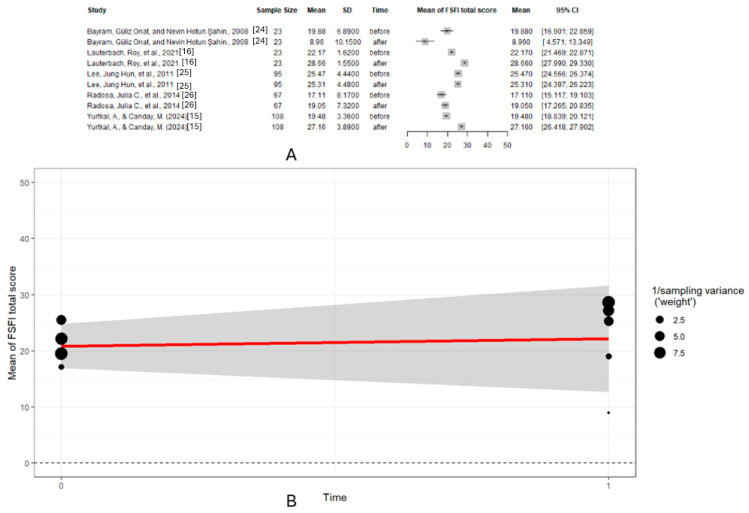
Change in sexual quality of life following VH. (**A**) Forest plot presenting study-specific mean FSFI total scores before and after VH, with corresponding 95% confidence intervals. (**B**) Bubble plot illustrating the pooled change in FSFI total score over time (0 = preoperative, 1 = postoperative). Bubble size is proportional to study weight (inverse sampling variance). The red line represents the estimated pooled effect, and the shaded area indicates the 95% confidence interval [15,16,24,25,26].

For vaginal hysterectomy (VH), the pooled mean baseline FSFI total score was 20.85 (95% CI, 16.9–24.79; *p* < 0.0001). The pooled mean change in FSFI total score over follow-up was 1.3 points (95% CI, −7.34–9.95; *p* = 0.71), indicating no statistically significant postoperative change in sexual function. [15,16,23,25,26] (Figure 7).

## 4. Discussion

### 4.1. Principal Findings

In this systematic review and meta-analysis, hysterectomy was not associated with a clinically meaningful improvement in sexual function across surgical approaches. Across all hysterectomy techniques analyzed, pooled mean FSFI scores indicated sexual dysfunction at baseline and remained below the established clinical cutoff (≤26.55) throughout follow-up. Changes in FSFI over time were small and were neither statistically nor clinically significant. Importantly, no differences in sexual function outcomes were observed between abdominal, laparoscopic, or vaginal hysterectomy. Taken together, these findings suggest that hysterectomy has a largely neutral effect on overall sexual quality of life and that expectations of postoperative sexual alterations should not guide surgical approach selection or decisions regarding cervical preservation.

### 4.2. Comparison with Existing Literature

Sexual function is a major component of quality of life and a key concern for women considering hysterectomy [28,29]. Expectations of postoperative sexual improvement, particularly related to symptom relief or preservation of pelvic anatomy, often influence patient preferences and surgical decision-making [30]. At the same time, surgeons may consider cervical preservation or minimally invasive approaches based on the assumption that these techniques better maintain sexual function [31]. However, the available evidence has been inconsistent, largely due to heterogeneity in study populations, surgical indications, outcome measures, and follow-up timing. This uncertainty complicates preoperative counselling and may contribute to unrealistic patient expectations.

Our findings align with and extend prior evidence evaluating sexual function after hysterectomy. Earlier systematic reviews often pooled heterogeneous questionnaires or analyzed a single postoperative time point despite substantial variability in follow-up intervals. In contrast, the present analysis prioritized validated instruments, predominantly the FSFI and applied longitudinal modelling to account for repeated postoperative assessments over time, enabling interpretation relative to clinically meaningful thresholds.

Dedden et al. found that hysterectomy, whether total or subtotal, was not associated with significant pre-to-post change in overall sexual function, regardless of surgical route, and emphasized that many patients continued to report sexual dysfunction after surgery [29]. Our findings closely corroborate these conclusions, and our route-first, instrument-focused synthesis further strengthens the interpretability of the message that hysterectomy is largely “sexually neutral” across surgical approaches.

Earlier randomized evidence summarized in the Cochrane review by Lethaby et al. likewise demonstrated no significant differences in sexual satisfaction or dyspareunia between total and subtotal hysterectomy, despite theoretical assumptions regarding the functional role of the cervix [32]. Together, the cumulative evidence indicates that neither surgical route nor cervical preservation has a meaningful impact on postoperative sexual function.

Kazemi et al. reported that hysterectomy for benign disease was not associated with a significant overall difference in sexual function compared with non-hysterectomy controls, although substantial heterogeneity was observed across instruments, populations, and surgical characteristics. While their control–comparator approach differs (hysterectomy vs. non-hysterectomy) from our pre–post framework, it suggests that average changes in sexual function after hysterectomy are small and inconsistent across contexts [33].

### 4.3. Synthesis of the Quantitative and Qualitative Evidence

#### 4.3.1. Laparoscopic Hysterectomy

Laparoscopic hysterectomy, including both TLH and LSH, was the most frequently studied minimally invasive approach in the present review. In the quantitative synthesis, neither TLH nor LSH was associated with a statistically or clinically significant alteration in postoperative sexual function. Across studies by Radosa et al., Bastu et al., Skorupska et al., Beyan et al., Kayataş et al., and Yurtkal et al., pooled FSFI total scores remained below the clinical cutoff for normal sexual function throughout follow-up [14,15,19,22,26,27].

Qualitative evidence from studies not eligible for meta-analysis supported these findings. Zimmermann et al. reported an improvement in FSFI total scores among women younger than 35 years undergoing laparoscopic hysterectomy; however, outcomes were not stratified by laparoscopic subtype, limiting conclusions regarding TLH versus LSH [34]. Shiber et al. observed improvements in FSFI total and pain subdomain scores following TLH, but the small sample size and lack of a comparator group restrict generalizability [35].

Additional studies using non-validated questionnaires reported mixed short-term changes in sexual satisfaction and pain following laparoscopic hysterectomy, without evidence of sustained improvement. Kafy et al. described a decline in satisfaction and pain scores following both TLH and supracervical laparoscopic hysterectomy [36]. Collectively, both quantitative and qualitative evidence suggests that laparoscopic hysterectomy does not confer a consistent advantage in sexual function compared with other surgical approaches.

#### 4.3.2. Abdominal Hysterectomy

Total abdominal hysterectomy was evaluated in multiple studies included in the meta-analysis. Across studies by Can et al., Beyan et al., Kürek Eken et al., El-Bassioune et al., Kayataş et al., and Bayram et al., pooled FSFI and ASEX scores demonstrated no statistically significant improvement in postoperative sexual function, with baseline values generally falling within the range of sexual dysfunction and remaining below the clinical cutoff during follow-up [18,19,20,21,22,24].

Qualitative evidence further supports these findings. Earlier observational studies by Gütl et al. and Bayram et al. reported declines or minimal changes in sexual function following abdominal hysterectomy [24,37]. Comparisons between abdominal and laparoscopic approaches by Beyan et al. and Kürek Eken et al. similarly failed to demonstrate a consistent sexual function advantage for either route [19,20]. Johannesson et al. evaluated long-term sexual function outcomes following total abdominal, total laparoscopic, and robot-assisted total laparoscopic hysterectomy but did not perform direct comparisons between surgical routes. At 36 months postoperatively, they reported a mean decrease of 3.6 points in FSFI total score, and at 60 months they observed a mean improvement of 0.2 points in FSFI total score relative to the original baseline, suggesting that sexual function may remain impaired or decline over extended follow-up, irrespective of surgical technique [38,39]. These results suggest that although abdominal hysterectomy is more invasive, it does not result in worse sexual outcomes but likewise does not lead to clinically meaningful improvement.

#### 4.3.3. Vaginal Hysterectomy

Vaginal hysterectomy demonstrated outcomes comparable to those observed with laparoscopic and abdominal approaches. In the quantitative synthesis, studies by Radosa et al., Lauterbach et al., Lee et al., Bayram and Şahin, and Bastu et al. showed no statistically significant improvement in FSFI total scores over time, with postoperative values remaining below the threshold for normal sexual function [16,24,25,26,27].

Qualitative studies provide additional nuance. Kokanalı et al. reported a modest improvement in PISQ-12 total scores following vaginal hysterectomy, suggesting possible benefits in populations with pelvic floor symptoms [40]. In contrast, Gütl et al. observed declines in TSST scores following both vaginal and abdominal hysterectomy [37]. Schiavi et al. reported improvements in both FSFI and PISQ-12 scores after vaginal hysterectomy, highlighting the influence of questionnaire selection and domain emphasis on reported outcomes [41].

Overall, the available evidence indicates that vaginal hysterectomy does not result in a consistent or clinically meaningful improvement in sexual function compared with other approaches.

#### 4.3.4. Radical Hysterectomy

Radical hysterectomy was included in the review but excluded from quantitative synthesis due to limited study numbers and oncologic heterogeneity. Qualitative evidence suggests that sexual function outcomes are variable and influenced by extent of interventions (reduction in vaginal length) nerve-sparing techniques, adjuvant therapy (mainly radiation therapy and vaginal brachytherapy), and psychological and psychosocial factors.

Novackova et al. reported a modest decline in FSFI total scores following nerve-sparing radical hysterectomy [42], whereas Jiang et al. observed greater declines in FSFI scores over time [43]. In contrast, Carter et al. reported improvements in FSFI scores during longer follow-up after radical hysterectomy, suggesting partial recovery of sexual function [44]. These findings underscore the complexity of sexual outcomes in oncologic populations and support separate evaluation of radical hysterectomy from procedures performed for benign indications, thus warranting an unmet need of sexual counselling in this subgroup of patients.

Firmeza et al. reported a small decline in mean FSFI total score over a five-month follow-up period after hysterectomy performed for cervical cancer-related indications; however, the surgical route was not specified, limiting route-specific interpretation [45].

#### 4.3.5. Hysterectomy with Bilateral Salpingo-Oophorectomy

Studies including concomitant bilateral salpingo-oophorectomy BSO were excluded from quantitative synthesis to minimize endocrine heterogeneity; however, qualitative evidence provides important insights into the impact of ovarian removal on sexual function. Observational studies consistently indicate that hysterectomy with BSO is associated with a higher risk of postoperative sexual dysfunction than hysterectomy with ovarian preservation.

Celik et al. and Doğanay et al. reported declines in FSFI total scores following hysterectomy with BSO, independent of surgical route, suggesting that endocrine effects outweigh anatomical factors in determining postoperative sexual outcomes [46,47]. Similarly, Aziz et al. found no sexual function benefit associated with BSO using the McCoy Female Sexuality Questionnaire [48].

Overall, the available evidence supports treating hysterectomy with BSO as a distinct clinical entity. The abrupt hormonal changes associated with surgical menopause likely play a dominant role in postoperative sexual dysfunction, justifying the exclusion of studies including BSO from quantitative analysis and underscoring the importance of careful preoperative counselling. According to the latest guidelines, prophylactic salpingectomy does not seem to significantly impair ovarian function, endocrine function, or fertility; thus, its alignment with BSO is not necessary.

### 4.4. Cervical Preservation and Broader Quality-of-Life Considerations

Preservation of the cervix during hysterectomy, with the maintenance of the vaginal apex suspension and innervation, has long been hypothesized to improve postoperative sexual function and broader quality-of-life outcomes and has therefore influenced both patient preference and surgical decision-making. However, neither the findings of the present review nor the cumulative evidence from randomized and observational studies support a clinically meaningful advantage of subtotal over total hysterectomy with respect to sexual function.

Randomized trials by Ellström et al. and Kuppermann et al. demonstrated no significant differences in sexual function outcomes between total and subtotal hysterectomy across multiple domains, including desire, arousal, satisfaction, and pain [49,50]. Similarly, Dedden et al. reported no meaningful pre-to-post change in overall sexual function attributable to cervical preservation, irrespective of surgical route [51]. Observational data from Aziz et al. further failed to identify any protective effect of retaining the cervix on postoperative sexual outcomes [38]. Although Ferhi et al. and Radosa et al. both reported on postoperative improvement in FSFI total score value after subtotal hysterectomy [12,26]. Importantly, these findings were consistent across laparoscopic and abdominal, reinforcing the absence of a route-specific benefit associated with cervical preservation.

Beyond sexual function, proposed advantages of cervical preservation in other quality-of-life domains have also proved limited. While Aleixo et al. reported reduced intraoperative blood loss with laparoscopic supracervical hysterectomy, the absolute differences were small and unlikely to be clinically meaningful, whereas postoperative vaginal bleeding was more frequent following cervical preservation and may negatively affect patient satisfaction or require additional intervention. [52] Evidence synthesized in the Cochrane review by Lethaby et al. similarly demonstrated no significant differences between total and subtotal hysterectomy in urinary, bladder, or bowel outcomes [35].

Importantly, cervical preservation entails long-term considerations, including the risk of cervical stump bleeding and the potential for malignant transformation, as well as the requirement for continued cervical cancer screening [35]. In the absence of demonstrable benefits in sexual or overall quality of life, these factors represent a clinically relevant burden for patients and healthcare systems. Accordingly, current clinical practice guidelines, including those issued by the French College of Gynecologists and Obstetricians, recommend removal of the cervix during hysterectomy for benign indications when feasible [53]. The findings of the present study provide additional support for these recommendations.

### 4.5. Interpretation of Persistent Dysfunction

An important observation of the present analysis is that baseline FSFI values were already within the range of sexual dysfunction and remained below the normal threshold throughout follow-up. This suggests that sexual dysfunction in women undergoing hysterectomy is multifactorial and not solely attributable to uterine pathology. Consequently, postoperative sexual outcomes should be interpreted in the context of this pre-existing dysfunction, as hysterectomy may not fully reverse the factors contributing to impaired sexual function. These findings highlight the importance of preoperative assessment and counselling regarding sexual health expectations following surgery.

Previous studies have suggested that improvements observed in some cohorts may reflect relief of bleeding, pain, or bulk symptoms rather than direct effects of the surgical procedure itself. Our findings indicate that, at the population level, such symptom relief does not translate into normalization of overall sexual function. These results highlight the importance of recognizing sexual dysfunction as an independent clinical issue that may require targeted management, including hormonal evaluation, treatment of dyspareunia, psychological support, and sexual-, physiotherapy-, and relationship-focused interventions [54,55].

### 4.6. Strengths and Limitations

The principal strength of this systematic review and meta-analysis lies in the inclusion of the largest pooled dataset available to date addressing sexual function outcomes after hysterectomy. The study was conducted in accordance with a prospectively registered PROSPERO protocol, ensuring methodological transparency and minimizing the risk of selective reporting. By comparing all commonly employed surgical routes of hysterectomy, we aimed to provide the most comprehensive synthesis of the existing evidence.

Several limitations merit consideration. Although the FSFI offered consistency and clinical interpretability, reliance on a single instrument restricted the inclusion of studies using alternative validated questionnaires. Most included studies were observational, increasing susceptibility to residual confounding. In addition, postoperative assessment timing was inconsistently reported, with a substantial proportion of studies lacking precise follow-up intervals. While longitudinal modelling and sensitivity analyses were employed to address this limitation, the findings should be interpreted as reflecting average postoperative changes rather than precise time-dependent trajectories. Finally, the wide confidence intervals around several estimates indicate limited precision, particularly for less frequently studied surgical subtypes.

The indication of the surgery can greatly influence expected change in sexual function; we were unable to stratify the results based on indications. To take this problem into account and to improve transparency we listed the indications from the included studies in the Supplementary Material. Furthermore, studies assessing radical hysterectomy were included only in the qualitative synthesis to minimize clinical heterogeneity related to oncologic surgery and its associated psychological and treatment-related factors.

### 4.7. Conclusions and Implications

Hysterectomy is unlikely to result in clinically meaningful improvement or worsening in overall sexual function, regardless of surgical route or cervical preservation. Consequently, expectations of improved sexual quality of life should not solely guide decisions on surgical approach selection. Given the high prevalence of persistent sexual dysfunction, systematic assessment and proactive management of sexual health should be integrated into both preoperative counselling and postoperative care with the implementation of evidence-based local and international guidelines and well-established patient pathways to warrant availability.

## 5. Conclusions

Hysterectomy, regardless of surgical route or cervical preservation, appears to have a largely neutral effect on sexual quality of life. Preservation of the cervix does not confer measurable benefit and may expose patients to additional long-term risks. Surgical decision-making should therefore focus on the combination of factors such as safety, indication, and patient-specific factors as well as expectations of improved sexual function.

## Figures and Tables

**Table 1:** Characteristics of all included studies.

Study	No. of Patients	BMI ^1^	Type of Study ^2^	Type of Operation	Type of Questionnaire	Pre-Operative Total Score Value	Postoperative Result, Without Exact Time Notation	Postoperative Result 3 Months	Postoperative Result 5 Months	Postoperative Result 6 Months	Post Operative Result 12 Months	Postoperative Result 18 Months	Postoperative Result 24 Months	Postoperative Result 36 Months
Ferhi M, Marwen N, Abdeljabbar A, et al. 2024	30	N/A	Prospective cohort study	Supracervical	FSFI ^3^	13.2	21.1	N/A	N/A	N/A	N/A	N/A	N/A	N/A
Ferhi M, Marwen N, Abdeljabbar A, et al. 2024	30	N/A	Prospective cohort study	Supracervical	ASEX ^4^	24.6	20.1	N/A	N/A	N/A	N/A	N/A	N/A	N/A
Ferhi M, Marwen N, Abdeljabbar A, et al. 2024	30	N/A	Prospective cohort study	TAH ^5^	FSFI	10.7	17.5	N/A	N/A	N/A	N/A	N/A	N/A	N/A
Ferhi M, Marwen N, Abdeljabbar A, et al. 2024	30	N/A	Prospective cohort study	TAH	ASEX	25.5	21.9	N/A	N/A	N/A	N/A	N/A	N/A	N/A
Ferguson et al., 2024	198	25.1	RCT ^8^	TAH	FSFI	27.1	N/A	26.74	N/A	27.11	27.45	N/A	27.07	27.8
Ferguson et al., 2024	207	25.1	RCT ^8^	RH ^9^	FSFI	27.6	N/A	25	N/A	26.14	27.02	N/A	27.59	27.62
Skorupska, K., Wawrysiuk, S., 2021	47	27.8	Prospective	TAH	FSFI	22.8	N/A	N/A	N/A	N/A	21.8	N/A	N/A	N/A
Skorupska, K., Wawrysiuk, S., 2021	28	27.8	Prospective	TLH	FSFI	28.8	N/A	N/A	N/A	N/A	20.2	N/A	N/A	N/A
Skorupska, K., Wawrysiuk, S., 2021	103	27.8	Prospective	VH	FSFI	23	N/A	N/A	N/A	N/A	22.1	N/A	N/A	N/A
Skorupska, K., Wawrysiuk, S., 2021	221	27.8	Prospective	Supracervical	FSFI	25.6	N/A	N/A	N/A	N/A	26.3	N/A	N/A	N/A
Yurtkal, A., & Canday, M. (2024)	84	N/A	prospective cohort study	TAH	FSFI	21.84	24.36	N/A	N/A	N/A	N/A	N/A	N/A	N/A
Yurtkal, A., & Canday, M. (2024)	88	N/A	prospective cohort study	TLH	FSFI	21.94	25.67	N/A	N/A	N/A	N/A	N/A	N/A	N/A
Yurtkal, A., & Canday, M. (2024)	108	N/A	prospective cohort study	VH	FSFI	19.48	27.16	N/A	N/A	N/A	N/A	N/A	N/A	N/A
Lauterbach, Roy, et al., 2021	23	29.4	prospective cohort study	VH	FSFI	22.17	28.66	N/A	N/A	N/A	N/A	N/A	N/A	N/A
Kiyak, Huseyin, et al., 2021	72	29.6	RCT ^8^	TAH	FSFI	20.75	N/A	18.4	N/A	N/A	N/A	N/A	N/A	N/A
Can, Ö. K., & ÖT Güler, Ö. T. (2020).	56	N/A	prospective study	TAH	FSFI	15.78	20.51	N/A	N/A	N/A	N/A	N/A	N/A	N/A
Can, Ö. K., & ÖT Güler, Ö. T. (2020).	56	N/A	prospective study	TAH	ASEX	18.46	17.66	N/A	N/A	N/A	N/A	N/A	N/A	N/A
Beyan, Emrah, et al., 2020	96	46.5	observational cohort study	TAH	FSFI	18.46	19.35	N/A	N/A	N/A	N/A	N/A	N/A	N/A
Beyan, Emrah, et al., 2020	259	46.5	observational cohort study	TLH	FSFI	17.89	21.65	N/A	N/A	N/A	N/A	N/A	N/A	N/A
Eken, Meryem Kürek, et al., 2016	42	29.05	prospective cohort study	TAH	ASEX	16.1	12.9	N/A	N/A	N/A	N/A	N/A	N/A	N/A
Eken, Meryem Kürek, et al., 2016	42	29.05	prospective cohort study	TLH	ASEX	17.2	10.8	N/A	N/A	N/A	N/A	N/A	N/A	N/A
Mahmoud, Ahmed Adel, et al., 2020	30	N/A	Analytical prospective study	TAH	FSFI	30.24	N/A	N/A	N/A	30.66	N/A	N/A	N/A	N/A
Mahmoud, Ahmed Adel, et al., 2020	30	N/A	Analytical prospective study	TLH	FSFI	30.12	N/A	N/A	N/A	31	N/A	N/A	N/A	N/A
Kayataş, Semra, et al., 2017	31	N/A	prospective observational study	TAH	FSFI	21.7	23.2	N/A	N/A	N/A	N/A	N/A	N/A	N/A
Kayataş, Semra, et al., 2017	31	N/A	prospective observational study	TAH	ASEX	17.4	15.2	N/A	N/A	N/A	N/A	N/A	N/A	N/A
Kayataş, Semra, et al., 2017	35	N/A	prospective observational study	TLH	FSFI	21.6	23.6	N/A	N/A	N/A	N/A	N/A	N/A	N/A
Kayataş, Semra, et al., 2017	35	N/A	prospective observational study	TLH	ASEX	16.3	15.9	N/A	N/A	N/A	N/A	N/A	N/A	N/A
Sukgen, Gökmen, and Aşkı Ellibeş Kaya., 2018	28	N/A	prospective cohort study	TLH	FSFI	27.2	N/A	N/A	N/A	N/A	28.9	N/A	N/A	N/A
Bayram, Güliz Onat, and Nevin Hotun Şahin., 2008	70	N/A	Prospective comparative, descriptive study	TAH	FSFI	22.27	15.96	N/A	N/A	N/A	N/A	N/A	N/A	N/A
Bayram, Güliz Onat, and Nevin Hotun Şahin., 2008	23	N/A	Prospective comparative, descriptive study	VH	FSFI	19.88	8.96	N/A	N/A	N/A	N/A	N/A	N/A	N/A
Lee, Jung Hun, et al., 2011	95	24.93	prospective consecutive observational study	VH	FSFI	25.47	25.31	N/A	N/A	N/A	N/A	N/A	N/A	N/A
Radosa, Julia C., et al., 2014	98	27.2	Observational cohort study	TLH ^6^	FSFI	18.27	20.45	N/A	N/A	N/A	N/A	N/A	N/A	N/A
Radosa, Julia C., et al., 2014	67	27.2	Observational cohort study	VH ^7^	FSFI	17.11	19.05	N/A	N/A	N/A	N/A	N/A	N/A	N/A
Bastu, Ercan, et al., 2016	34	27.4	RCT	TLH	FSFI	17.65	N/A	13.71	N/A	N/A	N/A	N/A	N/A	N/A
Bastu, Ercan, et al., 2016	36	27.4	RCT	VH	FSFI	16.11	N/A	11.89	N/A	N/A	N/A	N/A	N/A	N/A
Studies not included in the statistical analysis														
Zimmermann, Julia SM, et al., 2023	73	N/A	prospective cohort study	N/A	FSFI	19.25	24.15	N/A	N/A	N/A	N/A	N/A	N/A	N/A
Shiber, L-DJ, et al., 2015	80	N/A	Prospective observational study	TLH	FSFI	22.8 (median)	25.8 (median)	N/A	N/A	N/A	N/A	N/A	N/A	N/A
Kafy, Souzan, et al., 2009	40	N/A	retrospective study	Supracervical	not validated questionnaires	N/A ^14^	N/A	N/A	N/A	N/A	N/A	N/A	N/A	N/A
Kafy, Souzan, et al., 2009	40	N/A	retrospective study	TLH	not validated questionnaires	N/A ^14^	N/A	N/A	N/A	N/A	N/A	N/A	N/A	N/A
Gütl, P., et al., 2002	44	N/A	prospective study	TAH	TSST ^13^	12.89	N/A	11.76	N/A	N/A	N/A	N/A	N/A	11.56
Gütl, P., et al., 2002	46	N/A	prospective study	VH	TSST	12.38	N/A	11.31	N/A	N/A	N/A	N/A	N/A	11.02
Johannesson U. et al., 2023	162	N/A	prospective cohort study	N/A	FSFI	25.2	N/A	N/A	N/A	25.3	23.6	N/A	21.6	N/A
Forsgren, C., Amato, M., & Johannesson, U. (2022).	242	26.4	prospective cohort study	N/A	FSFI	17.9	N/A	N/A	N/A	21	19.1	N/A	N/A	N/A
Kokanalı, Mahmut Kuntay, et al., 2015	58	30	prospective observational study	VH	PISQ-12	29.52	31.98	N/A	N/A	N/A	N/A	N/A	N/A	N/A
Schiavi, Michele Carlo, et al., 2018	414	25.7	retrospective study	VH	PISQ-12 ^10^	30.51	37.1	N/A	N/A	N/A	N/A	N/A	N/A	N/A
Novackova, Marta, et al., 2022	65	N/A	prospective study	RH	FSFI	26.46	25.67	N/A	N/A	N/A	N/A	N/A	N/A	N/A
Jiang, Hongyuan, et al., 2016	216	N/A	prospective, non-randomized controlled clinical trial	RH	FSFI	24.48	18.9	N/A	N/A	N/A	N/A	N/A	N/A	N/A
Carter, Jeanne, et al., 2010	28	N/A	prospective study	RH	FSFI	15.72	N/A	20.58	N/A	23.66	25.44	25.01	22.63	N/A
Firmeza et al., 2024	71	N/A	prospective cohort study	RH	FSFI	17.22	N/A	N/A	16.19	N/A	N/A	N/A	N/A	N/A
Celik, Husnu, et al., 2008	37	27.6	prospective study	VH + BSO	FSFI	24.11	22.44	N/A	N/A	N/A	N/A	N/A	N/A	N/A
Celik, Husnu, et al., 2008	55	27.6	prospective study	TAH + BSO	FSFI	24.86	22.07	N/A	N/A	N/A	N/A	N/A	N/A	N/A
Doğanay, Melike, et al., 2019	78	28.5	retrospective study	N/A	FSFI	22.2	26	N/A	N/A	N/A	N/A	N/A	N/A	N/A
Doğanay, Melike, et al., 2019	82	28.2	retrospective study	N/A + BSO ^11^	FSFI	22.6	25.8	N/A	N/A	N/A	N/A	N/A	N/A	N/A
Aziz, Adel, et al., 2005	217	N/A	Prospective, observational study	N/A	MFSQ ^12^	55.41	54.61	N/A	N/A	N/A	N/A	N/A	N/A	N/A
Aziz, Adel, et al., 2005	106	N/A	Prospective, observational study	N/A + BSO	MFSQ	52.63	51.86	N/A	N/A	N/A	N/A	N/A	N/A	N/A
Kuppermann, Miriam, et al., 2005	68	N/A	RCT	Supracervical	not validated questionnaires	N/A ^14^	N/A	N/A	N/A	N/A	N/A	N/A	N/A	N/A
Kuppermann, Miriam, et al., 2005	67	N/A	RCT	TAH	not validated questionnaires	N/A ^14^	N/A	N/A	N/A	N/A	N/A	N/A	N/A	N/A
Ellström Engh, Marie A., Karin Jerhamre, and Karin Junskog., 2010	52	25.9	RCT	TAH	MFSQ	48.2	48.6	N/A	N/A	N/A	N/A	N/A	N/A	N/A
Ellström Engh, Marie A., Karin Jerhamre, and Karin Junskog., 2010	50	25.9	RCT	Supracervical	MFSQ	47.2	48.6	N/A	N/A	N/A	N/A	N/A	N/A	N/A

^1^ Body Mass Index. ^2^ The type of the included study. ^3^ Female Sexual Function Index. ^4^ Arizona Sexual Experience Scale. ^5^ Total abdominal hysterectomy. ^6^ Total laparoscopic hysterectomy. ^7^ Vaginal hysterectomy. ^8^ randomized controlled trial. ^9^ Radical hysterectomy. ^10^ Pelvic organ prolapse/Urinary Incontinence Sexual Questionnaire. ^11^ bilateral salpingo-oophorectomy. ^12^ McCoy Female Sexuality Questionnaire ^13^ Tübinger scale for sexual therapy ^14^ Total score could not be defined due to using a not validated questionnaire.

## Data Availability

The data used in this systematic review and meta-analysis were extracted from previously published studies. The extracted dataset and analytic code are available from the corresponding author upon reasonable request.

## Supplementary Materials

The following supporting information can be downloaded at: https://www.mdpi.com/article/10.3390/medsci14030396/s1, Figure S1: Risk of bias among RCT-s using the Rob 2 tool; Figure S2: Risk of bias among non-RCT using the ROBINS-I tool; Table S1: The assessment of certainty of evidence using the GRADE tool; Table S2: The indications for surgery in studies included [12,13,14,15,16,17,18,19,20,21,22,23,24,25,26,27,28,29,30,31,32,33,34,35,36,37,38,39,40,41,42,43,44,45,46,47,48,49,50]. Table S3: ISSM MOOSE Checklist, Table S4: PRISMA 2020 abstract checklist, Table S5: PRISMA 2020 checklist.

## Abbreviations

The following abbreviations are used in this manuscript:

ASEX	Arizona Sexual Experience Scale
BSO	Bilateral salpingo-oophorectomy
FSFI	Female Sexual Function Index
GRISS	The Golombok–Rust Inventory of Sexual Satisfaction
LSH	Laparoscopic subtotal hysterectomy
MFSQ	McCoy Female Sexuality Questionnaire
PICO	Population–intervention–comparison–outcome
PISQ-12	Pelvic organ prolapse/Urinary Incontinence Sexual Questionnaire
PRISMA	Preferred Reporting Items for Systematic Reviews and Meta-Analyses
QoL	Quality of life
TAH	Total abdominal hysterectomy
TLH	Total laparoscopic hysterectomy
TSST	Tübinger scale for sexual therapy
VH	Vaginal hysterectomy
