# Supplementary material for: Assessment of Sexual Function Following Hysterectomy: A Systematic Review and Meta-Analysis
Source: Med Sci (Basel). 2026 Jul 16;14(3):396. doi: 10.3390/medsci14030396 (PMC13413434; doi:10.3390/medsci14030396)
Supplement: Supplementary file 1 [file medsci-14-00396-s001.zip › medsci-4339852 Table S1 GRADE.pdf]

	Certainty assessment							Effect			
Intervention	No. of studies	Type of studies	Risk of bias	Inconsistency	Indirectness	Imprecision	Other considerations	No. of patients	Absolute (95%CI)	Certainty	Importance
Change in FSFI total score											
TAH vs TLH	7	Randomised and nonrandomised studies	Not serious	Not serious	Not serious	Not serious	Downgraded due to marked imbalance in participant numbers between intervention arms, which may bias the pooled effect estimates.	847	-0.3926 vs 5.2747	Moderate	Critical
TAH vs VH	9	Randomised and nonrandomised studies	Not serious	Not serious	Not serious	Not serious	Downgraded due to marked imbalance in participant numbers between intervention arms, which may bias the pooled effect estimates.	683	-4.8214 vs 7.884	Moderate	Critical
TLH vs VH	7	Randomised and nonrandomised studies	Not serious	Not serious	Not serious	Not serious	Downgraded due to marked imbalance in participant numbers between intervention arms, which may bias the pooled effect estimates.	796	-0,5947 vs 6.745	Moderate	Critical
Change in FSFI total score/month											
TAH vs TLH	6	Randomised and nonrandomised studies	Not serious	Not serious	Not serious	Not serious	Downgraded due to marked imbalance in participant numbers between intervention arms, which may bias the pooled effect estimates.	467	-0,0113 vs -0,1921	Moderate	Critical
