# Supplementary material for: Assessment of Sexual Function Following Hysterectomy: A Systematic Review and Meta-Analysis
Source: Med Sci (Basel). 2026 Jul 16;14(3):396. doi: 10.3390/medsci14030396 (PMC13413434; doi:10.3390/medsci14030396)
Supplement: Supplementary file 1 [file medsci-14-00396-s001.zip › medsci-4339852 Table S2 Supplementary_indications.pdf]

Ferhi M, Marwen N, Abdeljabbar A, et al. 2024 [12]	Uterine leiomyomas	Urogenital prolapses	Adenomyosis					
Ferguson et al., 2024 [13]	Low-Risk Early-Stage Cervical Cancer							
Skorupska, K., Wawrysiuk, S., 2021 [14]	fibroids,	abnormal uterine bleeding	Pelvic organ prolapse					
Yurtkal, A., & Canday, M. (2024) [15]	AUB resistant to medical treatment	Pelvical organ prolapse	Myoma Uteri	Adenomyosis	Endometrial premalignant lesions	Cervical premalignant lesions		
Lauterbach, Roy, et al., 2021[16]	Pelvical organ prolapse							
Kiyak, Huseyin, et al., 2021[17]	Abnormal uterine bleeding	Myoma uteri	Adenomyosis	CIN	Endometrial hyperplasia			
Can, Ö. K., & ÖT Güler, Ö. T. (2020). [18]	Uterine leiomyomas	benign endometrial hyperplasia	Abnormal uterine bleeding	Fibroid	Ovarian cysts	postmenopausal bleeding		
Beyan, Emrah, et al., 2020 [19]	Myoma uteri	Adenomyosis	CIN 3	Dysfunctional uterine bleeding	Endometrial hyperplasia			
Eken, Meryem Kürek, et al., 2016 [20]	Endometrial hyperplasia	Cervical pathology	Myoma Uteri	Abnormal uterine bleeding				
Mahmoud, Ahmed Adel, et al., 2020 [21]	Uterine leiomyomas	Abnormal uterine bleeding	Adenomyosis	Cervical pathology				
Kayataş, Semra, et al., 2017 [22]	Benign conditions							
Sukgen, Gökmen, and Aşkı Ellibeş Kaya., 2018 [23]	Treatment resistant menometroragia	Endometrial hyperplasia	Myoma Uteri					
Bayram, Güliz Onat, and Nevin Hotun Şahin., 2008 [24]	Myoma uteri	Stress incontinence	pelvic relaxation	pelvic relaxation				
Lee, Jung Hun, et al., 2011 2011 [25]	Menorrhagia	Dysmenorrhea	Dysfunctional uterine bleeding	Chronic pelvic pain	Palpable abdominal mass	Abnormal cervical pathology		
Radosa, Julia C., et al., 2014 [26]	Benign uterine disorders							
Bastu, Ercan, et al., 2016 [27]	Benign conditions							
Zimmermann, Julia SM, et al., 2023 [34]	Symptomatic uterine fibroids	Endometriosis	Descensus uteri					

Shiber, L-DJ, et al., 2015 [35]	Benign conditions							
Kafy, Souzan, et al.. 2009 [36]	Severe menorrhagia	Fibroid uteri	Chronic pelvic pain					
Gütl, P., et al., 2002 [37]	Fibroid uteri	Ovarian cysts	Severe menstrual bleeding	Uterine prolapse	Ovarian tumours			
Johannesson U. et al., 2023 [38]	Benign conditions							
Forsgren, C., Amato, M., & Johannesson, U. (2022).[39]	Benign conditions							
Kokanalı, Mahmut Kuntay, et al., 2015 [40]	Pelvic organ prolapse							
Schiavi, Michele Carlo, et al., 2018 [41]	Pelvical organ prolapse							
Novackova, Marta, et al., 2022 [42]	Cervical cancer							
Jiang, Hongyuan, et al., 2016 [43]	Cervical cancer							
Carter, Jeanne, et al., 2010 [44]	early-stage cervical cancer							
Firmeza et al., 2024 [45]	Cervical cancer	Benign conditions						
Celik, Husnu, et al., 2008 [46]	Benign conditions							
Doğanay, Melike, et al., 2019 [47]	Uterine myoma	Abnormal uterine bleeding	Pelvic organ prolapse	Ovarian cyst				
Aziz, Adel, et al., 2005 [48]	Benign conditions							
Kuppermann, Miriam, et al., 2005 [49]	Severe menorrhagia	Leiomyoma	Pelvic or bladder pressure	Urinary incontinence				
Ellström Engh, Marie A., Karin Jerhamre, and Karin Junskog., 2010 [50]	Dysfunctional bleeding	Uterine leiomyoma	Dysmenorrhea/pelvic pain	Pelvic pain	Mechanical symptoms			

Supplementary table S2. The table shows the indications from the included studies. All indications are cited as in the original study
