# Supplementary material for: Assessment of Sexual Function Following Hysterectomy: A Systematic Review and Meta-Analysis
Source: Med Sci (Basel). 2026 Jul 16;14(3):396. doi: 10.3390/medsci14030396 (PMC13413434; doi:10.3390/medsci14030396)
Supplement: Supplementary file 1 [file medsci-14-00396-s001.zip › medsci-4339852 Table S4 PRISMA_flowchart_kesz.pdf]

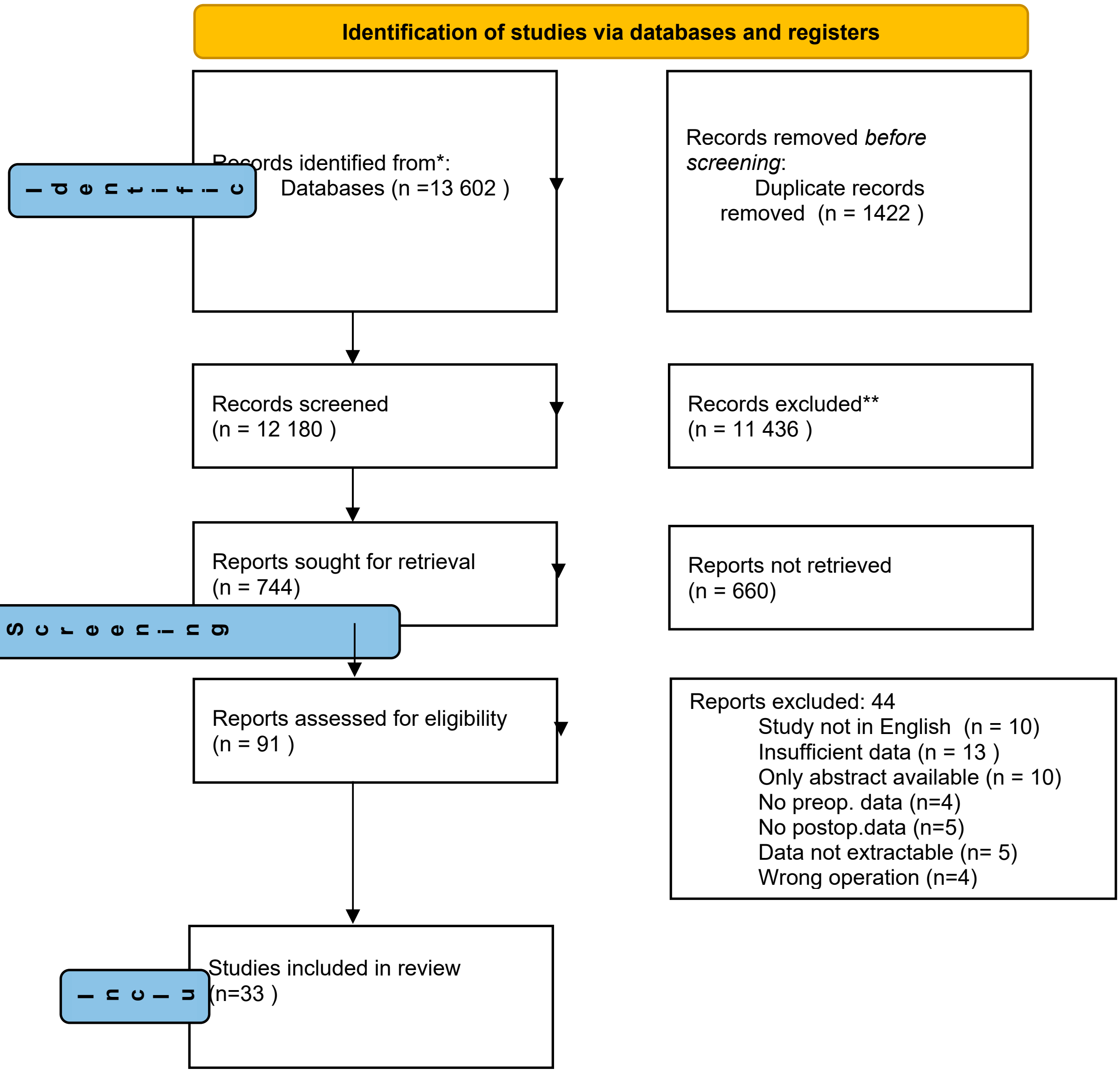
Identification of studies via databases and registers
Identific
Records identified from*:
Databases (n =13 602)
Records removed before screening:
Duplicate records removed (n = 1422)
Records screened
(n = 12 180)
Records excluded**
(n = 11 436)
Reports sought for retrieval
(n = 744)
Reports not retrieved
(n = 660)
Screening
Reports assessed for eligibility
(n = 91)
Reports excluded: 44
Study not in English (n = 10)
Insufficient data (n = 13)
Only abstract available (n = 10)
No preop. data (n=4)
No postop.data (n=5)
Data not extractable (n= 5)
Wrong operation (n=4)
Inclu
Studies included in review
(n=33)
