# Supplementary figures and images for: Assessment of Sexual Function Following Hysterectomy: A Systematic Review and Meta-Analysis
Source: Med Sci (Basel). 2026 Jul 16;14(3):396. doi: 10.3390/medsci14030396 (PMC13413434; doi:10.3390/medsci14030396)

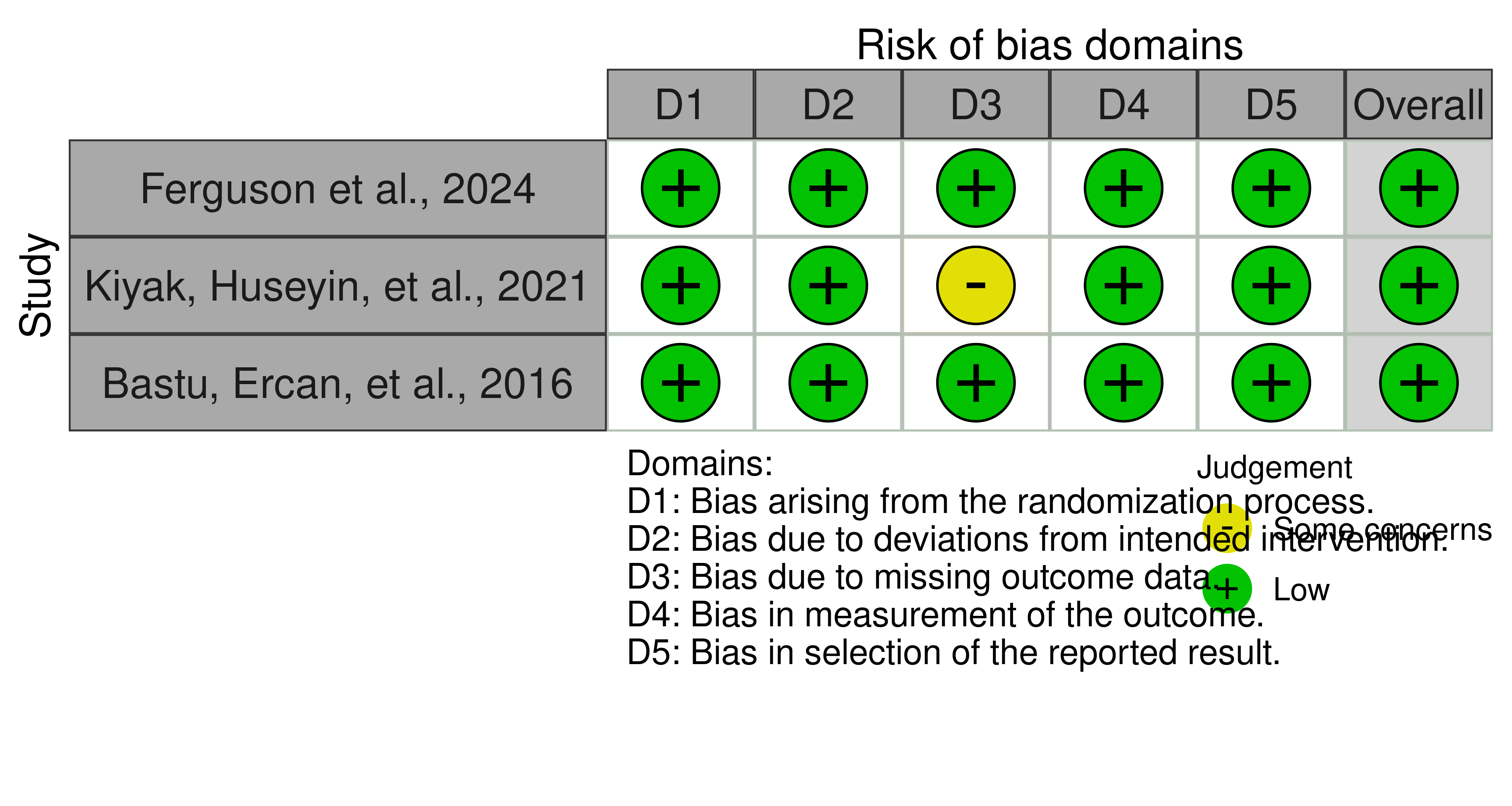

Supplement: Supplementary file 1 [file medsci-14-00396-s001.zip › medsci-4339852 Figure S1 ROB2.png]

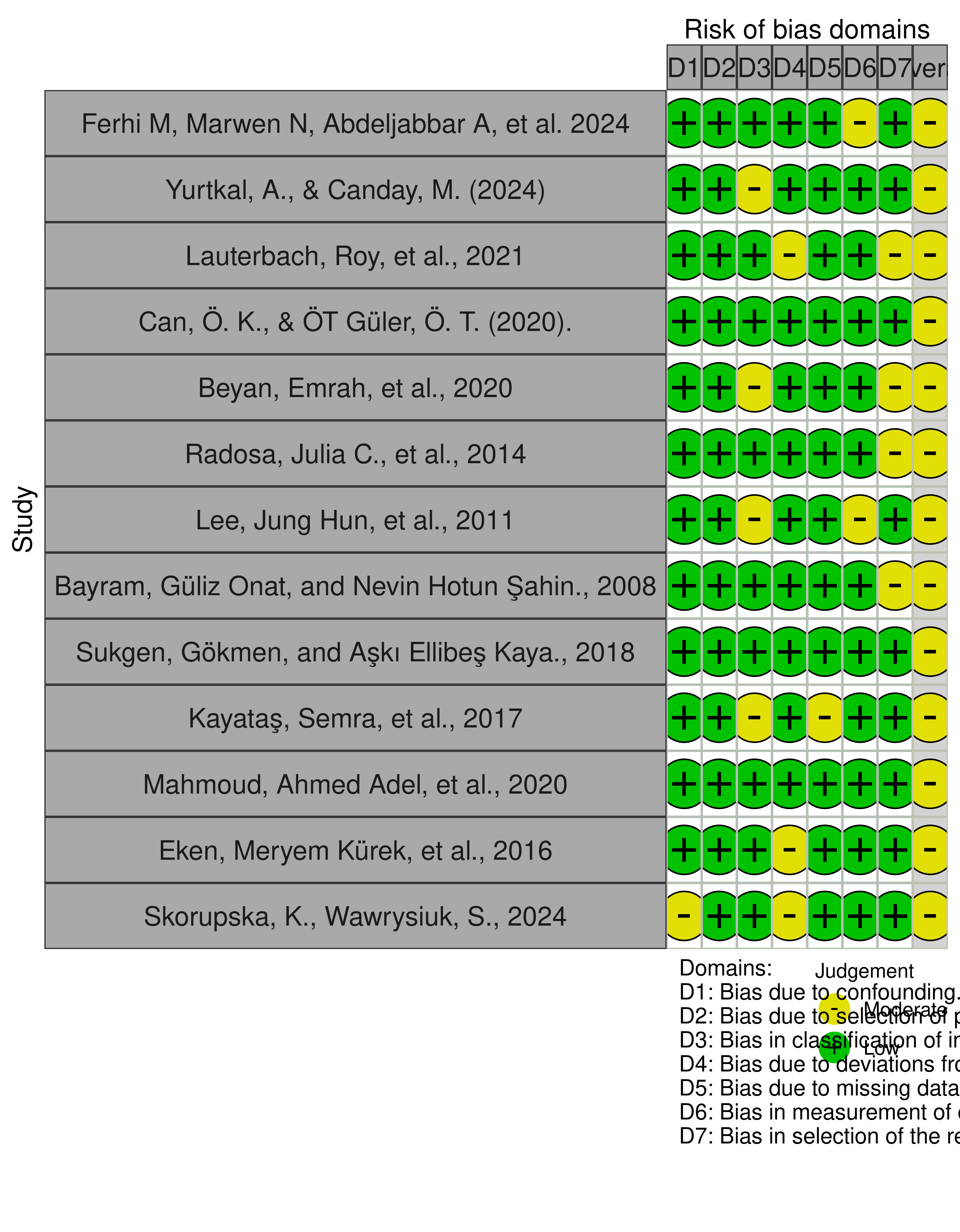

Supplement: Supplementary file 1 [file medsci-14-00396-s001.zip › medsci-4339852 Figure S2 ROBINS-I.png]
